# A Conductance-Based Silicon Synapse Circuit

**DOI:** 10.3390/biomimetics7040246

**Published:** 2022-12-16

**Authors:** Ashish Gautam, Takashi Kohno

**Affiliations:** Institute of Industrial Science, The University of Tokyo, Tokyo 153-8505, Japan

**Keywords:** biomimetic synapse circuit, shunting inhibition, synaptic reversal potential, adaptive STDP, spike pattern detection, neuromorphic computing, synaptic resolution

## Abstract

Neuron, synapse, and learning circuits inspired by the brain comprise the key components of a neuromorphic chip. In this study, we present a conductance-based analog silicon synapse circuit suitable for the implementation of reduced or multi-compartment neuron models. Compartmental models are more bio-realistic. They are implemented in neuromorphic chips aiming to mimic the electrical activities of the neuronal networks in the brain and incorporate biomimetic soma and synapse circuits. Most contemporary low-power analog synapse circuits implement bioinspired “current-based” synaptic models suited for the implementation of single-compartment point neuron models. They emulate the exponential decay profile of the synaptic current, but ignore the effect of the postsynaptic membrane potential on the synaptic current. This dependence is necessary to emulate shunting inhibition, which is thought to play important roles in information processing in the brain. The proposed circuit uses an oscillator-based resistor-type element at its output stage to incorporate this effect. This circuit is used to demonstrate the shunting inhibition phenomenon. Next, to demonstrate that the oscillatory nature of the induced synaptic current has no unforeseen effects, the synapse circuit is employed in a spatiotemporal spike pattern detection task. The task employs the adaptive spike-timing-dependent plasticity (STDP) learning rule, a bio-inspired learning rule introduced in a previous study. The mixed-signal chip is designed in a Taiwan Manufacturing Semiconductor Company 250 nm complementary metal oxide semiconductor technology node. It comprises a biomimetic soma circuit and 256 synapse circuits, along with their learning circuitries.

## 1. Introduction

The human brain has been officially designated as the most complex object encountered in our known universe. The endeavor to “understand” it is complemented by the dream of designing an intelligent machine. The research fields of neuroscience and artificial intelligence (AI) are dedicated to these “understanding” and “application” goals, respectively. One of the neuroscientific approaches is a bottom-up approach based on the mathematical modeling of the brain’s elementary computational units—neuronal cells, and synapses. Models using this approach simulate neuronal circuitries designed based on observed connectivity patterns or to specifically test a hypothesis, thereby improving our understanding of the neuronal pathway(s) in question [1,2]. However, this approach is not scalable, and runs into computational problems as the size of neuronal circuitry increases. In pursuit of the “application” goal, artificial neural network (ANN)-based deep learning models currently dominate the field of machine intelligence and have achieved human-level performance in tasks such as image classification and game playing [3,4]. They are inspired by the brain but represent the electrical activity of neuronal cells in an abstract sense. Spiking neural networks (SNNs), in contrast, are much more similar to the brain. These third-generation neural networks use spiking neuron models similar to those used in neuroscience studies. They represent information using spike timings or spike rates. They have been demonstrated to perform well in various benchmark tasks, such as spike pattern classification and image classification [5,6,7], and their performance approaches that of ANNs [8,9]. Moreover, with neuromorphic hardware, SNNs are more power-efficient than ANNs.

Neuromorphic researchers have designed dedicated hardware platforms that mimic and/or are inspired by the computational architecture of the brain. These chips (generally fabricated using complementary metal oxide semiconductor (CMOS) technology) primarily comprise silicon neurons and synapse circuits for emulating the electrical activity of the neuronal cells and synapses in the brain. One of their goals is to deepen our understanding of the brain through the real-time emulation of neuronal circuits. They can replicate the electrical activity of neuronal cells in real time via the implementation of multi-compartment neuron models [10,11] that are computationally expensive to simulate. In addition to modeling the electrical behavior of the soma (as is done in single-compartment neuron models), multi-compartment models also incorporate the spatiotemporal structures of dendritic trees by modeling them as separate compartments. These compartments are connected via resistors, mimicking the spatial profile of the cell being modeled. Reduced-compartment neuron models are a trade-off between the single-compartment point neuron model (that completely ignore dendritic computation) and detailed multi-compartment models. They generally have fewer compartments (two to four) and ignore the detailed spatial structures of the dendritic trees. They aim to replicate neuronal dynamics at the soma by capturing the somatodendritic interactions occurring in neuronal cells [12,13,14].

Neuromorphic chips that mimic neuronal activity use biologically plausible neuron and synapse circuits. Many such silicon neuron circuits (conductance-based or qualitatively modeled) have been implemented in low-power analog very large-scale integration-based neuromorphic chips [15,16,17]. In contrast, most low-power analog synapse circuits implement a “current-based” synapse model rather than a conductance-based one. The former ignores the effect of the postsynaptic membrane potential on the synaptic current. This dependence is incorporated in conductance-based models and is critical to emulate the phenomenon of shunting inhibition, which is thought to play important roles in information processing in the brain [18,19,20]. Shunting inhibition involves blocking the transmission of excitatory or inhibitory synaptic signals without strongly influencing the membrane potential of the soma. The interaction between the excitatory and shunting inhibitory synapses at different locations on the dendritic tree has also been suggested to enhance computational capability by realizing non-linear operations between their currents [19], or by virtual compartmentalization of the cell [20].

Implementing of a conductance-based synapse circuit requires a resistor-like circuit to incorporate the effect of the postsynaptic membrane potential on the synaptic current. In a few neuromorphic chips, the resistor-like circuit is implemented either using a transconductance amplifier [21,22,23] or switched-capacitor circuits [24,25]. However, these circuits operate in the above-threshold domain (of the metal oxide semiconductor (MOS) transistor), and thus consume a relatively high amount of power. In the subthreshold domain (of the MOS transistor), a low-power synapse circuit partially incorporates the desired effect of the postsynaptic membrane potential by using a transconductance-like circuit at the output stage of a differential pair integrator (DPI) synapse [26]. However, this implementation is not biologically plausible because the circuit does not implement a resistor-like element, and thus cannot reverse the polarity of the induced synaptic current necessary to replicate shunting inhibition. In [27], a subthreshold transconductance circuit was used to implement the resistor-like element. However, because of fabrication mismatch, it induced a high leakage current (static current when the circuit is inactive) into the post-synaptic node that disturbs the spiking dynamics of the soma circuit. Compensation for this leakage current required additional circuits that increase the overall power consumption of the neuron.

To address this gap, we propose a new low-power conductance-based synapse circuit and demonstrate the shunting inhibition on the fabricated chip. The required resistor-like element is designed using an oscillator and a switching capacitor-like circuit. It addresses the issues in the circuits described above. This circuit is intended for neuromorphic implementation of low-power biologically realistic neuronal networks in reduced or multi-compartment configurations. To demonstrate that its oscillatory output current has a similar effect on information processing as a non-oscillatory synapse circuit, it is applied to a spike pattern detection task [28,29] that is a very basic, biologically realistic information processing model. The same task was solved by non-oscillatory synapse circuits [30] and our results empirically show that both oscillatory and non-oscillatory synapse circuits have similar performance.

The remainder of this manuscript is organized as follows. The next section begins with a description of the phenomenon of shunting inhibition, followed by the details of the synapse, neuron, and learning circuits and the spike pattern detection task. Three experimental results, the characteristics of the proposed synapse circuit, a demonstration of shunting inhibition, and the spike pattern detection task are presented in the Results section. Finally, a discussion of the results and conclusions derived from this study are provided.

## 2. Materials and Methods

### 2.1. Shunting Inhibition

Based on a detailed kinetic model of synaptic transmission [31], a phenomenological model of the postsynaptic current in a neuronal cell can be described as follows:

(1) $$I_{\mathrm{syn}}\left(t\right)=g_{\mathrm{syn}}\left(t\right)\cdot \left(V_{\mathrm{mem}}\left(t\right)-E_{\mathrm{syn}}\right),$$

where $V_{\mathrm{mem}}$, $g_{\mathrm{syn}}$, and $E_{\mathrm{syn}}$ are the postsynaptic membrane potential, synaptic conductance, and synaptic reversal potential, respectively. This description is phenomenological, and in the relevant voltage ranges, the induced synaptic current exhibits an approximately linear dependence on the difference between $V_{\mathrm{mem}}$ and $E_{\mathrm{syn}}$. In the simplest models, the time-dependent synaptic conductance $g_{\mathrm{syn}}$ (t) has a bi-exponential profile similar to that of an alpha function. From Equation (1), it can be seen that the polarity of the synaptic current induced in the neuronal cells depends not only on the synaptic receptor involved (that fixes $E_{\mathrm{syn}}$), but also on the instantaneous $V_{\mathrm{mem}}$. Synapses with their $E_{\mathrm{syn}}$ significantly higher (lower) than the resting $V_{\mathrm{mem}}$ have excitatory (inhibitory) effects; upon activation, they depolarize (hyperpolarize) the postsynaptic neuronal cell membrane. Synapses with their $E_{\mathrm{syn}}$ close to the resting $V_{\mathrm{mem}}$ are called shunting inhibitory synapses. Most contemporary low-power analog synapse circuits [26,32] implement a current-based synapse model that mimics the bi-exponential profile of the synaptic current, but ignores its dependence on the difference between the instantaneous $V_{\mathrm{mem}}$ and $E_{\mathrm{syn}}$. As is clear from Equation (1), the circuit implementation requires a resistor-like element between $V_{\mathrm{mem}}$ and$\;E_{\mathrm{syn}}$.

**Figure 1:**
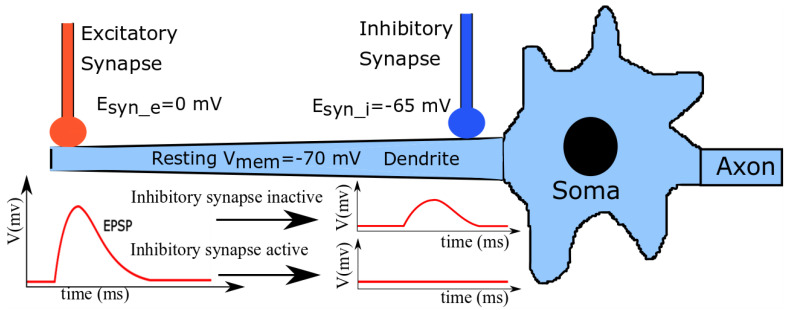
A schematic illustration of a neuronal cell with excitatory and shunting inhibitory synapses. The EPSP generated by excitatory synapse attenuates as it reaches the soma.

A simplified schematic illustration of a neuronal cell is shown in Figure 1. A group of excitatory and shunting inhibitory synapses distal and proximal to the soma impinge on a dendritic branch. In line with generally measured neurophysiological values [33], the resting *V*_mem_ of the cell, synaptic reversal potential of the excitatory synapses ($E_{\mathrm{syn}\_e}$), and that of shunting inhibitory synapses ($E_{\mathrm{syn}\_i}$) are approximately −70 mV, 0 mV (typical for α-amino-3-hydroxy-5-methyl-4-isoxazolepropionic acid (AMPA) synapses), and −65 mV (typical for GABAa (γ-aminobutyric acid type A) synapses), respectively. Upon the standalone activation of the excitatory synapses, the induced current depolarizes the local membrane and drives its potential towards$\;E_{\mathrm{syn}\_e}$. This disturbance travels as a gradually attenuating excitatory postsynaptic potential (EPSP) towards the soma and causes it to spike (if the depolarizing current is strong enough). The standalone activation of the shunting inhibitory synapses has a negligible depolarization effect on the neuronal membrane, but increases the local membrane conductance around the soma (as$\;E_{\mathrm{syn}\_i}$ is close to$\;V_{\mathrm{mem}}$). However, when both excitatory and shunting inhibitory synapses are activated together, the EPSPs generated by the excitatory synapses are attenuated by the shunting inhibitory synapses when the traveling EPSPs reach near the soma. If the inhibition is strong enough, the depolarization induced by the excitatory synapses is completely silenced and the soma’s membrane potential remains undisturbed; this phenomenon is called shunting inhibition.

The relative spatial placements and temporal activations of the shunting inhibitory and excitatory synapses on dendritic arbors are believed to play a significant role in the computational capabilities of neuronal cells. In previous studies [19,33], it was shown that shunting inhibition implements an approximate multiplication between the excitatory and inhibitory synaptic conductance within the dendritic tree. In another study [20], it was demonstrated that in a neuronal cell, the shunting inhibitory synapses can modify the number of electrically isolated dendritic compartments; these then act as independent compartments for detecting the coincidence of incoming spikes. A study discussing the escape behavior of crayfish [18] hypothesized and then experimentally confirmed how the relative positioning and activation of excitatory and shunting inhibitory synapses on a neuronal cell can help fish decide whether to initiate an escape response or continue usual feeding activity in response to a potentially dangerous situation. Thus, implementing the shunting inhibitory synapse circuits is necessary to replicate the electrical behavior of the neuronal circuits in the brain. Moreover, given their potential to enhance neuronal computation via specific non-linear interactions with excitatory inputs, it is evident that low-power shunting inhibitory synapse circuits can add to the capabilities of neuromorphic chips implementing multi-compartment or reduced-compartment neuron models.

### 2.2. Conductance-Based Synapse Circuit

**Figure 2:**
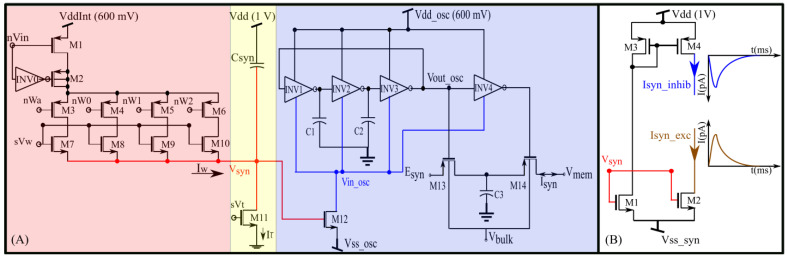
(**A**) Conductance-based synapse circuit with an oscillator-based transconductance stage. (**B**) Output stage of current-based excitatory and inhibitory synapse circuit with the same digital-to-analog converter (DAC) and integrator as the first two stages.

A schematic of the proposed synapse circuit is shown in Figure 2A. It has three stages: a digital-to-analog converter (DAC, M1–M10), an integrator stage ($C_{\mathrm{syn}}$ and M11) similar to the log domain integrator (LDI), and a transconductance stage (M12–M14, C1–C3, and INV1–INV4). The DAC and integrator stages are similar to the synapse circuit proposed in our previous study [27,34].

In the DAC, M7–M10 are binary-weighted transistors. The bias voltage $sV_{w}$ controls the strength of the synaptic current, and the switches M3–M6 configure the four-bit synaptic efficacy. The efficacy is stored in digital memories updated by a learning circuitry. Upon the application of an input pulse (~2 ms wide) at the gate of M1, depending on the value of $sV_{w}$ and the synaptic efficacy, the DAC sources a current into the node V_syn_ and charges it for the duration of the input pulse. The inverter INV0 along with M2 is for reducing the charge injection effect. Once the input pulse is turned off, V_syn_ is discharged linearly by a constant current sunk by transistor M11, which operates in the saturation region (for V_syn_ > 4 U_T_). The bias voltage $sV_{t}$ and capacitance$\;C_{\mathrm{syn}}$ control the discharge rate. In most contemporary current-based synapse circuits, the node V_syn_ activates a MOS transistor (M2 in Figure 2B) for converting the linear voltage V_syn_ to an exponential current (I_syn_exc_), as shown in Figure 2B. In the proposed circuit, V_syn_ activates the transconductance stage.

The transconductance stage is designed using an unbalanced switched-capacitor-like circuit (INV4, M13, M14, and C3) activated by a ring oscillator-type circuit (INV1–3). In a typical ring oscillator, the source terminals of the PMOS and NMOS devices of the inverters ($V_{\mathrm{dd}\_\mathrm{osc}}$ and node $V_{\mathrm{in}\_\mathrm{osc}})$ are connected to constant voltage sources, and the circuit generates a pulsed waveform with the maximum and minimum values of $V_{\mathrm{dd}\_\mathrm{osc}}$ and$\;V_{\mathrm{in}\_\mathrm{osc}}$, respectively. The propagation time of the inverters determines the pulse width and frequency of the waveform. It is calculated as the average time taken by the inverter’s PMOS transistor to charge its output capacitance and that taken by the NMOS transistor to discharge the same capacitance. To derive this, we consider the inverter INV1 in Figure 2A. When its gate voltage$\;V_{g}$ is near $V_{\mathrm{dd}\_\mathrm{osc}}$ its NMOS transistor discharges its output node, as follows:

(2) $$I_{0\_NM}\cdot \exp\left(\frac{k_{n\_NM}\left(V_{g}\right)-V_{\mathrm{in}\_\mathrm{osc}}}{U_{T}}\right)\cdot \left(1-exp\left(\frac{-\left(V_{\mathrm{out}}-V_{\mathrm{in}\_\mathrm{osc}}\right)}{U_{T}}\right)\right)=-C_{\mathrm{out}}\cdot \frac{dV_{\mathrm{out}}}{dt},$$

where $\;I_{0\_NM}$ and $\;k_{n\_NM}$ are the current scaling factor and capacitive coupling ratio of the inverter’s NMOS transistor, respectively. $U_{T}$ is the thermal voltage. $V_{\mathrm{out}}\;$ is the output node of INV1. The body effect is ignored in the calculations. Separating the variables in Equation (2) and integrating yields as follows:

(3) $$\int_{V_{\mathrm{out}}\left(0\right)}^{V_{\mathrm{out}}\left(t\right)}\frac{dV_{\mathrm{out}}}{1-\exp\left(\frac{-\left(V_{\mathrm{out}}-V_{\mathrm{in}\_\mathrm{osc}}\right)}{U_{T}}\right)}=\frac{-I_{0\_NM}}{C_{\mathrm{out}}}\cdot exp\left(\frac{k_{n\_NM}\left(V_{g}\right)-V_{\mathrm{in}\_\mathrm{osc}}}{U_{T}}\right)\int_{0}^{t}d\tau .$$

By choosing the halfway point between $V_{\mathrm{dd}\_\mathrm{osc}}$ and $V_{\mathrm{in}\_\mathrm{osc}}$ to calculate the discharging time ($t_{PHL})$, the integral limit on the left-hand side ranges from $V_{\mathrm{initial}}\;$ to (Vinitial+Vin_osc)2 and that on the right-hand side ranges from 0 to $t_{PHL}.$ Solving Equation (3) for $t_{PHL}$ yields as follows:

(4) $$t_{PHL}=\frac{C_{\mathrm{out}}\cdot U_{T}}{I_{0\_NM}\cdot exp\left(\frac{k_{n\_NM}\left(V_{g}\right)-V_{\mathrm{in}\_\mathrm{osc}}}{U_{T}}\right)}\cdot ln\left\{\frac{1-exp\left(\frac{-\left(V_{\mathrm{initial}}-V_{\mathrm{in}\_\mathrm{osc}}\right)}{U_{T}}\right)}{exp\left(\frac{-\left(V_{\mathrm{initial}}-V_{\mathrm{in}\_\mathrm{osc}}\right)}{2U_{T}}\right)-exp\left(\frac{-\left(V_{\mathrm{initial}}-V_{\mathrm{in}\_\mathrm{osc}}\right)}{U_{T}}\right)}\right\}.$$

By repeating the same derivation for the charging process via the inverter’s PMOS transistor, the charging time is given as follows:

(5) $$t_{PLH}=\frac{C_{\mathrm{out}}\cdot U_{T}}{I_{0\_PM}\cdot exp\left(\frac{k_{p\_PM}(V_{\mathrm{dd}\_\mathrm{osc}}-V_{g})}{U_{T}}\right)}\cdot ln\left\{\frac{1-exp\left(\frac{-\left(V_{\mathrm{dd}\_\mathrm{osc}}-V_{\mathrm{initial}}\right)}{U_{T}}\right)}{exp\left(\frac{-\left(V_{\mathrm{dd}\_\mathrm{osc}}-V_{\mathrm{initial}}\right)}{2U_{T}}\right)-exp\left(\frac{-\left(V_{\mathrm{dd}\_\mathrm{osc}}-V_{\mathrm{initial}}\right)}{U_{T}}\right)}\right\},$$

where $\;I_{0\_PM}$ and $\;k_{p\_PM}$ are the current scaling factor and capacitive coupling ratio of the PMOS transistor in the inverter, respectively. In Equation (4), $t_{PHL}$ is calculated when $V_{g}\approx V_{\mathrm{dd}\_\mathrm{osc}}$ and $V_{\mathrm{initial}}\approx V_{\mathrm{dd}\_\mathrm{osc}}$. Similarly, in Equation (5), $t_{PLH}$ is calculated when $V_{g}\approx V_{\mathrm{in}\_\mathrm{osc}}$ and $V_{\mathrm{initial}}\approx V_{\mathrm{in}\_\mathrm{osc}}$. To simplify the equation, we assume that $I_{0\_PM}$=$I_{0\_NM}=I_{0},\;\;k_{n\_NM}=k_{p\_PM}=k$. Based on these substitutions, and considering that $V_{\mathrm{dd}\_\mathrm{osc}}-V_{\mathrm{in}\_\mathrm{osc}}>4U_{T}$, the propagation time of the inverter (the average of $t_{PLH}$ and $t_{PHL}$), is given as follows:

(6) $$t_{P}=\frac{1}{I_{0}\cdot exp\left(\frac{k\left(V_{\mathrm{dd}\_\mathrm{osc}}\right)-V_{\mathrm{in}\_\mathrm{osc}}}{U_{T}}\right)}\cdot \frac{C_{out}\cdot U_{T}\cdot \left(V_{\mathrm{dd}\_\mathrm{osc}}-V_{\mathrm{in}\_\mathrm{osc}}\right)}{4}.$$

**Figure 3:**
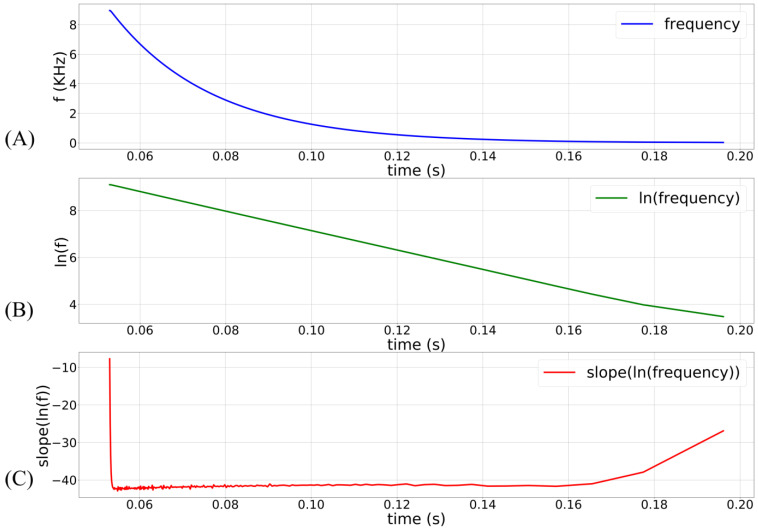
Transient of the oscillator’s frequency in a typical synaptic event. The circuit activates at 50 ms with $sV_{t}$ = 100 mV, $sV_{w}$ = 280 mV, and maximum synaptic efficacy. (**A**) Frequency plot of the oscillator’s output; (**B**) natural logarithm of the frequency plot in (**A**); (**C**) the slope of the plot in (**B**). Plots (**B**) and (**C**) show that the oscillator’s frequency decays exponentially in a typical synaptic event. The exponential dependence is lost when M11 comes out of the saturation region (V_syn_ < 4 *U_T_*).

**Figure 4:**
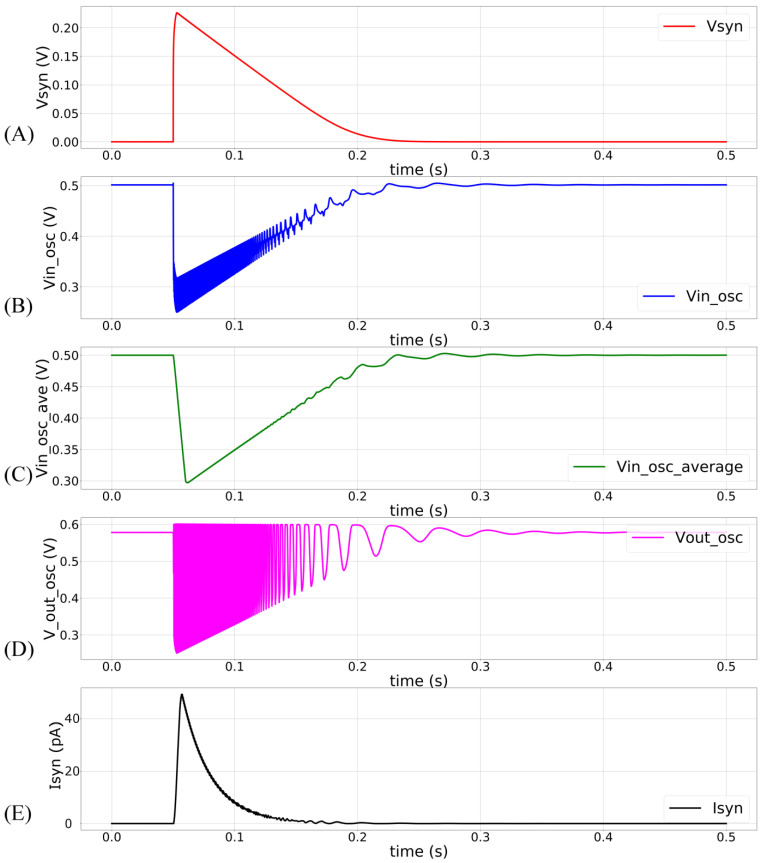
The synapse circuit is activated at 50 ms with $sV_{t}$ = 100 mV, $sV_{w}$ = 280 mV, and maximum synaptic efficacy. (**A**) Linearly discharging profile of V_syn_; (**B**) profile of V_in_osc_. Oscillations are owing to the current sourced out of the oscillator circuit; (**C**) moving average profile of the node V_in_osc_, plotted with a time window of 15 ms; (**D**) profile of the node V_out_osc_ showing oscillator’s output, where the amplitude of the oscillations decreases linearly; (**E**) the induced synaptic current plotted with a time window of 5 ms.

In the proposed circuit, only $V_{\mathrm{dd}\_\mathrm{osc}}$ is a constant-voltage source (600 mV). The node $V_{\mathrm{in}\_\mathrm{osc}}$ is not a voltage source. The current sourced and sunk by the inverters (INV1–4) and M12 determine its voltage. The propagation time ($t_{P}$) of the inverters (which controls the oscillator’s frequency and pulse width) is thus not constant, and the frequency has an exponential dependence on$\;V_{\mathrm{in}\_\mathrm{osc}}$ (see Figure 3). The terminal $V_{\mathrm{ss}\_\mathrm{osc}}$ is kept above 0 V (~35 mV) to minimize the leakage current via M12. This renders the oscillator circuit inactive when there is no input pulse. In this inactive state, V_syn_ and $V_{\mathrm{in}\_\mathrm{osc}}$ are close to 0 V and$\;V_{\mathrm{dd}\_\mathrm{osc}}$, respectively. The oscillator remains off because there is insufficient headroom for oscillation. In response to a pulse input to the DAC stage, the linearly charging and discharging V_syn_ activates M12 that sinks current out of $V_{\mathrm{in}\_\mathrm{osc}}$, pulling it down and activating the oscillator. The profiles of V_syn_ and $V_{\mathrm{in}\_\mathrm{osc}}$ upon circuit activation at 50 ms (obtained via Spectre simulation) are shown in Figure 4A,B. The voltage $V_{\mathrm{in}\_\mathrm{osc}}$ is approximately linearly related to V_syn_, as indicated by the moving average of $V_{\mathrm{in}\_\mathrm{osc}}$ (Figure 4C). The oscillator’s output (V_out_osc_) is shown in Figure 4D. The oscillator’s output V_out_osc_ activates the switched-capacitor-like circuit (M13, M14, INV4, and C3) that implements an asymmetric resistor-type element between $V_{\mathrm{mem}}$ and$\;E_{\mathrm{syn}}$. Here, $V_{\mathrm{mem}}$ is fixed at 600 mV. When inactive, the gates of M13 and M14 remain close to$\;V_{\mathrm{dd}\_\mathrm{osc}}$ and a very small current flows out of $\;V_{\mathrm{mem}}$ (if $\;E_{\mathrm{syn}}>V_{\mathrm{mem}}$). Upon circuit activation, M13 and M14 receive out-of-phase pulses (via INV4) whose amplitudes decrease from $V_{\mathrm{dd}\_\mathrm{osc}}$ to $\;V_{\mathrm{in}\_\mathrm{osc}}$. These pulses activate M13 and M14 in the subthreshold domain (where the drain current of the MOS device is exponentially related to its gate voltage). As the amplitude of these pulses decreases linearly over time (because $V_{\mathrm{in}\_\mathrm{osc}}$ increases linearly over time), an exponential current is induced out of $\;V_{\mathrm{mem}}$ (for $\;E_{\mathrm{syn}}>V_{\mathrm{mem}}$). Figure 4E plots the moving average profile of the induced synaptic current for $\;E_{\mathrm{syn}\;}=$ 700 mV and$\;V_{\mathrm{mem}}=$ 600 mV.

This circuit functions as a non-linear resistor. Unlike an ideal resistor, the induced current has an exponential dependence on the difference between $E_{\mathrm{syn}}$ and $\;V_{\mathrm{mem}}$. The current in a PMOS device increases with its source-gate overdrive voltage, and for a fixed $V_{\mathrm{dd}\_\mathrm{osc}}$, the overdrive in M13 and M14 is higher for $E_{\mathrm{syn}}>V_{\mathrm{mem}}\;$ than for $\;E_{\mathrm{syn}}<V_{\mathrm{mem}}$. Owing to the exponential current–voltage (I–V) relationship in the subthreshold domain, the resistance emulated is exponentially larger for values of $E_{\mathrm{syn}}$ < $V_{\mathrm{mem}}$ (in comparison with values of $E_{\mathrm{syn}}$ > $V_{\mathrm{mem}}$), leading to an “asymmetric” I–V relationship (See Section 3.1). The transconductance stage of the proposed circuit can also be used as a non-linear resistor between terminals $E_{\mathrm{syn}}$ and $\;V_{\mathrm{mem}}$ if the node V_syn_ is fixed at a constant value.

### 2.3. Architecture of Silicon Neuron Circuit

**Figure 5:**
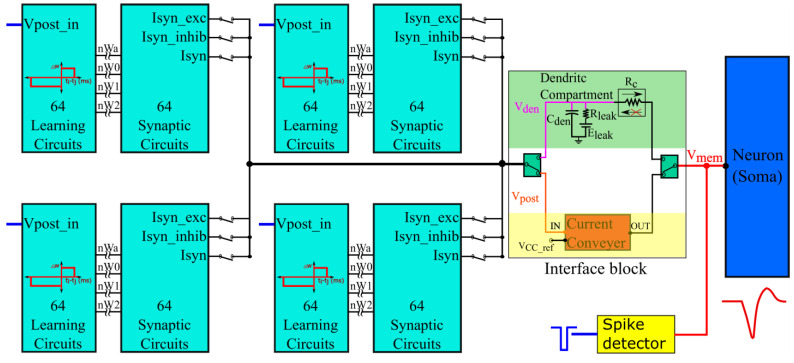
Block diagram representation of the neuron’s architecture. The current conveyor as a link implements a single-compartment point neuron model. It also fixes the voltage at node V_post_ equal to V_CC_ref_. The unidirectional resistor R_c_ as a link implements the unidirectional two-compartment model.

The synapse circuit described in Section 2.2 is incorporated into a silicon neuron circuit fabricated in the Taiwan Semiconductor Manufacturing Company (TSMC) 250 nm technology node. The block diagram is shown in Figure 5. It has 256 synapse circuits in groups of four (64 circuits per group) for activating a qualitatively modeled soma circuit [16,35]. The polarities of the synapse circuits can be configured as a group. In an excitatory or inhibitory configuration (I_syn_exc_ or I_syn_inhib_ terminals chosen as outputs in Figure 2 and Figure 5), the current-based synapse model is evoked. Here, the induced synaptic current does not depend on the postsynaptic potential, whereas it does in the conductance-based configuration (terminal I_syn_ is chosen as the output in Figure 2 and Figure 5). Upon activation, the synapse circuits induce a current into the soma circuit via an interface circuit, causing it to either depolarize or hyperpolarize. The spiking behavior and current polarity of the neuronal soma circuit are opposite to the convention. An excitatory (inhibitory) synapse circuit has $\;E_{\mathrm{syn}}$ lower (higher) than the resting $V_{\mathrm{mem}}$ and depolarizes (hyperpolarizes) the soma by sinking (sourcing) current out of (into) it, causing the postsynaptic membrane potential to drop (rise) below (above) its resting value. This is because the soma circuit is designed primarily using PMOS transistors with much smaller leakage currents than their NMOS counterparts. This minimizes the power consumption of the circuit. In this study, the soma circuit is configured in the fast-spiking Class 1 mode of Hodgkin’s classification (no spike-frequency adaptation). Its spikes are converted into pulses using a spike detector circuit (see [30] for details). Subsequently, these pulses are fed back to the learning circuitry (representing the postsynaptic spike, V_post_in_ in Figure 5). All synapse circuits have a learning circuitry to implement adaptive spike-timing-dependent plasticity (STDP) learning, which updates the synaptic efficacy based on the spike timings of the pre- and postsynaptic spikes. To perform pattern detection on-chip, input spike trains are transmitted from a PC to the chip via a field-programmable gate array. An on-chip spike address decoder circuit is used to activate the synapse circuits. The details of this spike transfer module can be found in a previous study [30].

#### 2.3.1. Interface Block

**Figure 6:**
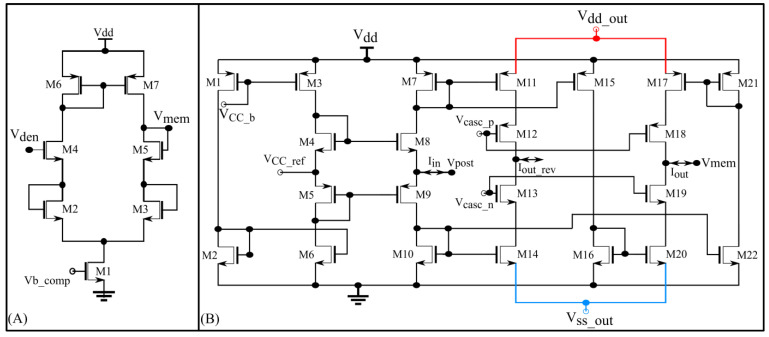
(**A**) Unidirectional resistor designed using a single-stage source degenerated transconductance circuit; (**B**) current conveyor circuit with two output branches. I_out_ (I_out_rev_) is used when the 256 synapse circuits are connected to V_post_ via terminal I_syn_exc_ (I_syn_).

The interface block has two circuits available as a link between the synaptic and soma circuits: a unidirectional resistor (green part in Figure 5) composed of a transconductance circuit (Figure 6A), and a bidirectional current conveyor circuit (yellow part in Figure 5) whose circuitry is shown in Figure 6B. The former configures the neuron as a unidirectional two-compartment neuron circuit, whereas the latter does so as a single-compartment point neuron circuit.

The unidirectional two-compartment neuron configuration was described in detail in a previous study [29]. It has a somatic compartment comprising a soma circuit and dendritic compartment comprising a leak resistor ($R_{\mathrm{leak}}$) and dendritic capacitor ($C_{\mathrm{den}}$) for integrating the synaptic current induced by the synapse circuits. The membrane potentials of the somatic and dendritic compartments are represented by V_mem_ and V_den_, respectively. Based on their potential difference, current flows into or out of the somatic compartment via a unidirectional resistor ($R_{c}$). As the name implies, no current flows into or out of the dendritic compartment via the unidirectional resistor as would occur in an ideal two-compartment neuron model. The dendritic capacitor $C_{\mathrm{den}}$ is approximately 8.5 pF. The leak resistor $R_{\mathrm{leak}}$ is implemented using one synapse circuit in the conductance-based configuration. This neuron configuration is used to demonstrate the shunting inhibition.

In the single-compartment neuron configuration, the membrane capacitance of the soma integrates the synaptic current. However, in the circuit implementation, if synapse circuits are connected directly to the soma circuit, their parasitic capacitance and leakage current disturb the spiking dynamics of the soma circuit. Hence, a bidirectional current conveyor circuit that replicates the current induced by the synapse circuits into the soma circuit is used as a link between them. Its two output branches induce currents with opposite polarities. I_out_ has the same polarity as I_in_ and I_out_rev_ has the opposite polarity. The current conveyor circuit is a current-controlled current source that fixes the node voltage common to the output terminals of all 256 synapse circuits (V_post_ in Figure 5 and Figure 6B) at a fixed value, approximately $\;V_{\mathrm{CC}\_\mathrm{ref}}$ = 600 mV. Thus, in this single-compartment configuration, the induced synaptic current depends only on the voltage parameters configuring the synapse circuit. As the node V_post_ is fixed to a constant value, the conductance-based synapse circuits act as excitatory synapse circuits (when connected to the soma circuit via the output terminal I_out_rev_ and with $\;E_{\mathrm{syn}}$ fixed higher than $\;V_{\mathrm{CC}\_\mathrm{ref}}$ or V_post_). In the experiments, the voltage bias $\;V_{\mathrm{CC}\_b}$ was 630 mV (370 mV below $V_{\mathrm{dd}}$). For relatively weaker bias voltages ($\;V_{\mathrm{CC}\_b}$ = 700 mV), the precise shapes and timings of the current induced by the synapse circuits were not conveyed to the soma circuit. However, with a stronger bias voltage$\;(V_{\mathrm{CC}\_b}$ = 630 mV) the circuit consumes relatively higher power and induces significant noise. The noise is due to thermal noise in silicon and the bias voltage source. The ripple noise of the power line was extremely low as ultralow ripple power supplies were used. The induced thermal noise caused the soma circuit’s resting membrane potential (when around 800 mV) to vary randomly by approximately 50 mV. This random variation was higher for resting membrane potential values close to the spiking threshold of the soma circuit (700 mV in single-compartment configuration). To minimize this noise, *V*_dd out_ and $V_{\mathrm{ss}\mathrm{out}}$ were fixed at 949 mV and 50 mV, respectively, i.e., smaller and larger than their ideal values of 1 and 0 V, respectively. This reduced the random variation to approximately 30 mV (at a resting membrane potential of 800 mV). Furthermore, to minimize the effect of thermal noise, the resting membrane potential of the soma circuit was increased to approximately 850 mV. The power consumed by this circuit was approximately 90 nW (measured in the Spectre simulation). In this configuration, the 256 conductance-based synapse circuits were connected to the soma circuit via the current conveyor’s output terminal I_out_rev_. The spike pattern detection was performed in this configuration to demonstrate that the switching nature of the conductance stage did not affect the performance of the task.

In terms of the complexity and biological plausibility, the unidirectional two-compartment model lies between the single- and two-compartment neuron models. The single-compartment neuron configuration cannot be used to demonstrate shunting inhibition, because the postsynaptic node (output terminals of the synapse circuits, V_post_) is fixed at a constant value by the feedback action of the current conveyor circuit. In this study, we present three experimental results, as summarized in Table 1.

#### 2.3.2. Learning Circuit

**Figure 7:**
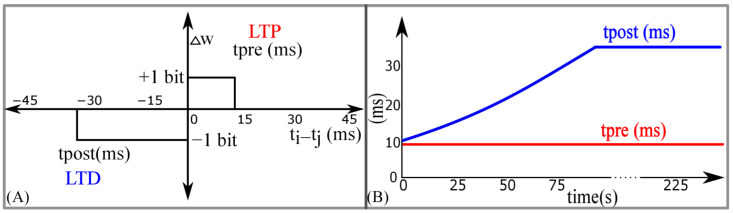
Adaptive spike-timing-dependent plasticity (STDP) learning. (**A**) Rectangular STDP learning rule; (**B**) adaptation of *t*_post_ in rectangular STDP learning rule during the learning process.

**Figure 8:**
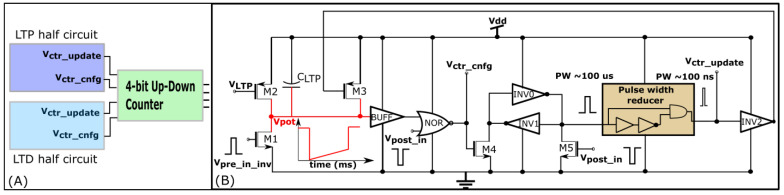
Learning circuitry. (**A**) Block diagram; (**B**) Long term potentiation (LTP) half-circuit.

Similar to STDP learning, the adaptive STDP learning rule updates the synaptic efficacy based on the time difference between the pre- and postsynaptic spikes. However, the update in the efficacy is restricted to ±1 bits. This ±1 bit update is encoded by the rectangular STDP learning function (Figure 7A) and is mathematically expressed as follows:

(7) $$\Delta w_{j}=\{\begin{array}{c} +1\;bit,\;\;\;\;if\;t_{j}\leq t_{i}\;\mathrm{and}\;t_{i}-t_{j}<t_{\mathrm{pre}}\;\mathrm{and}\;w<w_{\max}\;\;\;\;\left(LTP\right),\\ -1\;bit,\;\;\;\;if\;t_{j}>t_{i}\;\mathrm{and}\;t_{j}-t_{i}\;<t_{\mathrm{post}}\;\mathrm{and}\;w>w_{\min}\;\;\left(LTD\right), \end{array}\;$$

where $t_{\mathrm{pre}}$ is the maximum delay of the postsynaptic spike after the presynaptic spike leading to potentiation (LTP). $t_{\mathrm{post}}$ is the maximum delay of the presynaptic spike after the postsynaptic spike leading to depression (LTD); $t_{j}$ and $t_{i}$ represent the timing of the pre- and postsynaptic spikes, respectively. The efficacy saturates at its maximum ($w_{\max}$) and minimum ($w_{\min}$) values. The learning parameter $t_{\mathrm{pre}}$ is kept constant during learning and $t_{\mathrm{post}}$ is increased, as shown in Figure 7B. The details of the adaptive STDP learning are provided in a previous study [29]. Each synapse circuit has a learning circuit to implement the adaptive STDP learning. A block diagram is shown in Figure 8A. The synaptic efficacy is stored in a four-bit up-down counter and updated by the circuits controlling its LTP and LTD. A conceptual schematic of the half-circuit controlling the LTP of the synaptic efficacy is shown in Figure 8B. The details of the circuit operation are provided in another study [30]. The value of $V_{\mathrm{LTP}}$ (which controls$\;t_{\mathrm{pre}}$) was fixed at 780 mV. The initial value of $V_{\mathrm{LTD}}$ (that controls$\;t_{\mathrm{post}}$) was fixed at 783 mV and was adapted to higher values during learning (as shown in Figure 7B). The chip does not contain an adaptation circuitry, and the adaptation of $V_{\mathrm{LTD}}$ was controlled via an external voltage source.

#### 2.3.3. Spike Pattern Detection Task

The goal of the spike pattern detection task is to detect a 50 ms long spike pattern hidden within stochastic input spike trains at irregular intervals using a single neuron in an unsupervised manner. The neuron receives spike trains via $N_{\mathrm{aff}}$ synapses ($N_{\mathrm{aff}}$ is the number of afferents). These spike trains are generated independently via an inhomogeneous Poisson process. The instantaneous firing rate ranges between 0 Hz and 90 Hz (the minimum time period for changing from 0 Hz to 90 Hz is 50 ms). Each afferent spikes at least once in a 50 ms duration, fixing 20 Hz as the minimum spiking frequency. Upon the generation of stochastic spike train (with a length of 225 s), a random 50 ms long segment (the target spike pattern) is chosen and copied. Subsequently, the original spike train is segmented into 50 ms long sections. Depending on the required spike pattern repetition frequency (chosen as 25 or 10%), certain randomly chosen sections are replaced by the target spike pattern. Consecutive 50 ms sections are avoided in this copy–paste process. This process ensures that only the specific spike time of the afferents distinguishes the spike pattern. The population average spike rate (measured in 10 ms time bins) is approximately the same inside and outside the spike patterns (approximately 54 Hz). These spike trains are used as inputs with $N_{\mathrm{aff}}=$ 256 for the spike pattern detection experiment. The spike trains are 225 s long, and 50 runs were performed for each experimental setup.

The ideal STDP learning model has been shown to perform well in such spatiotemporal pattern detection tasks; however, its circuit implementation requires high-resolution synaptic efficacy [5,28]. In contrast, low-power circuits generally adopt memory devices under five bits for synaptic efficacy, owing to the silicon area and power constraints. Thus, we propose a hardware-friendly, bioinspired learning rule called adaptive STDP. In previous studies, the task described above was solved using adaptive STDP learning via simulations [29] and circuit experiments [30] with current-based four-bit excitatory synapses. In this study, we solved this task using the conductance-based four-bit synapse circuit described in Section 2.2.

## 3. Results

### 3.1. Characteristics of the Conductance-Based Synapse Circuit

**Figure 9:**
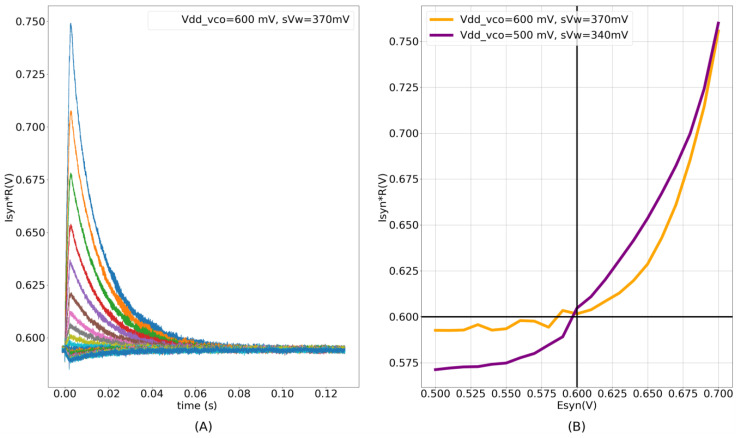
(**A**) Synaptic current measured as voltage for 21 different values of $E_{\mathrm{syn}}$ (500 mV to 700 mV) with $V_{\mathrm{mem}}$ = 600 mV. Each of the 21 measurements were repeated six times and ignorable standard deviation was observed among the repeated runs; (**B**) non-linear I–V relationship. The Orange trace plots the peak intensities of synaptic currents in (**A**) and the purple trace plots the same with $V_{\mathrm{dd}\_\mathrm{osc}}$ and $sV_{w}$ reduced to 500 mV and 340 mV, respectively. Both traces are plotted against corresponding values of $E_{\mathrm{syn}}$.

The experimental results for the fabricated conductance-based synapse circuit are shown in Figure 9. The induced synaptic current is in the picoampere range, and is measured as a voltage using an on-chip high-resistance circuit (a source-degenerated transconductance circuit similar to Figure 6A). The synaptic currents were measured for 21 different values of $\;E_{\mathrm{syn}}$ (from 500 to 700 mV) with $V_{\mathrm{mem}}$ = 600 mV and $V_{\mathrm{dd}\_\mathrm{osc}}$ = 600 mV. Six measurements were performed for each of the $E_{\mathrm{syn}}$ values. The time traces of one measurement are shown in Figure 9A. The mean peak intensities (averaged across six measurements) of the synaptic currents in Figure 9A for the $E_{\mathrm{syn}}$ values are plotted in Figure 9B, and show the non-linear I–V relationship (orange trace). A negligible standard deviation was observed across the repeated runs. The circuit was designed such that the static power consumption did not exceed 2 pW (across all of the process corners). The measured static power consumption of the single synapse circuit on the chip was approximately 1.64 pW. This was calculated by an on-chip measurement of the average static current drawn by 256 synaptic circuits. Owing to the transconductance stage, the dynamic power consumption of this circuit is higher than that of contemporary low-power current-based synapse circuits designed to operate in the subthreshold domain. The dynamic power consumption to generate an AMPA-type synaptic current is approximately 5.2 pJ/spike. The voltage parameters used for the measurements were as follows: $V_{\mathrm{dd}\_\mathrm{osc}}$ = 600 mV, $sV_{w}$ = 315 mV, $\;sV_{t}$ = 160 mV, $\;E_{\mathrm{syn}}$ = 700 mV, and $V_{\mathrm{mem}}$= 600 mV, with the maximum synaptic efficacy. These parameters were used in the spike pattern detection task (Section 3.3). The dynamic power consumption contribution from the DAC stage alone is approximately 60 fJ/spike. The DAC is active only during the input pulse (~2 ms). The majority of the dynamic power in the circuit is consumed by the transconductance stage, which is active both during the input pulse and the discharge phase of the synaptic current. The circuit voltage parameters used in these measurements are listed in Table 2. The parameter $sV_{t}$ =160 mV generated a synaptic current with small time constants (approximately 3–5 ms). For clarity in the plotted image, $sV_{t}$ = 80 mV was chosen for the measurements shown in Figure 9. In all the other measurements in this study, it was fixed at 160 mV.

To further characterize the circuit, the results measured using the Spectre simulator (unless stated otherwise) are also presented. With $V_{\mathrm{dd}\_\mathrm{osc}}$, $V_{\mathrm{mem}}$, and $E_{\mathrm{syn}}$ fixed at 600, 600, and 700 mV in the static condition (no spike input), respectively, the current flowing into the node $V_{\mathrm{mem}}\;$is under 20 fA. The current flows into (out of) the node $V_{\mathrm{mem}}$ if $E_{\mathrm{syn}}$ > $V_{\mathrm{mem}}$ $(E_{\mathrm{syn}}$ < $V_{\mathrm{mem}}$). The dynamic power consumption of the circuit with synaptic efficacy values of 1 and 15 and a time constant of 3 ms is estimated as under 700 fJ/spike and 4.5 pJ/spike, respectively. This value is about 15 % lower than the experimentally measured value and the difference is probably due to fabrication process variations. The efficacy values of 1 and 15 induce synaptic currents with a peak intensity of 10 pA and 68 pA, respectively, corresponding to the oscillator’s maximum frequency of 2.4 kHz and 17.7 kHz, respectively. The higher the oscillation frequency, the higher the dynamic power consumption. The dynamic power consumption of the circuit can be reduced by using a smaller $V_{\mathrm{dd}\_\mathrm{osc}}$ (relative to $E_{\mathrm{syn}}$), at the expense of a higher current flowing into its output terminal $\;V_{\mathrm{mem}}$ (for $E_{\mathrm{syn}}>V_{\mathrm{mem}}$). Lowering $V_{\mathrm{dd}\_\mathrm{osc}}$ increases the source-gate overdrive voltage for M13 and M14 (see Figure 2), and when operating in the subthreshold region, an exponentially larger current flows into $\;V_{\mathrm{mem}}$. With $V_{\mathrm{dd}\_\mathrm{osc}}$ = 500 mV and all other parameters remaining the same, the static current flowing into $\;V_{\mathrm{mem}}$ increases from 20 fA to approximately 325 fA. Upon activation, the induced current is also exponentially larger. The dynamic power consumption with synaptic efficacy values of 1 and 15 is estimated as under 600 fJ/spike and 3.6 pJ/spike, respectively, corresponding to the oscillator’s maximum frequency of 3 KHz and 20.1 KHz and synaptic currents of 26 pA and 180 pA, respectively. Thus, with a smaller $V_{\mathrm{dd}\_\mathrm{osc}}$, for approximately similar values of the oscillator frequency and power consumption, the induced current is exponentially larger. The purple trace (experimental measurement) in Figure 9B plots the measured peak intensity for 21 different values of $E_{\mathrm{syn}}$ (averaged over six measurements), similar to the orange trace, but with $V_{\mathrm{dd}\_\mathrm{osc}}$ reduced from 600 mV to 500 mV. As the current induced with a smaller $V_{\mathrm{dd}\_\mathrm{osc}}$ is larger, the parameter $sV_{w}$ was reduced from 370 mV to 340 mV to ensure that the measured voltage remains in the linear range of the high-resistance circuit. The circuit operates reliably across all of the process corners for $V_{\mathrm{dd}\_\mathrm{osc}}$ > 450 mV.

Thus, the power consumption of this circuit can be minimized at the expense of the static current flowing out of its output terminal (305 fA when $V_{\mathrm{dd}\_\mathrm{osc}}$ is reduced from 600 mV to 500 mV), which can be compensated for at the level of the dendrites or the soma. In addition, the static current (along with the intensity of the induced synaptic current) can be controlled using the back-gate voltages $\;V_{\mathrm{bulk}}$ of M13 and M14 (see Figure 2). In the measurements above, $\;V_{\mathrm{bulk}}$ was fixed at 1 V; increasing it reduces the static current flowing into $\;V_{\mathrm{mem}}$. With $V_{\mathrm{dd}\_\mathrm{osc}}$ = 500 mV, $\;E_{\mathrm{syn}}$ = 700 mV, and $\;V_{\mathrm{bulk}}$ = 1.2 V (increased from 1 V), the static current reduces from 325 fA to under 50 fA.

### 3.2. Shunting Inhibition on Chip

**Figure 10:**
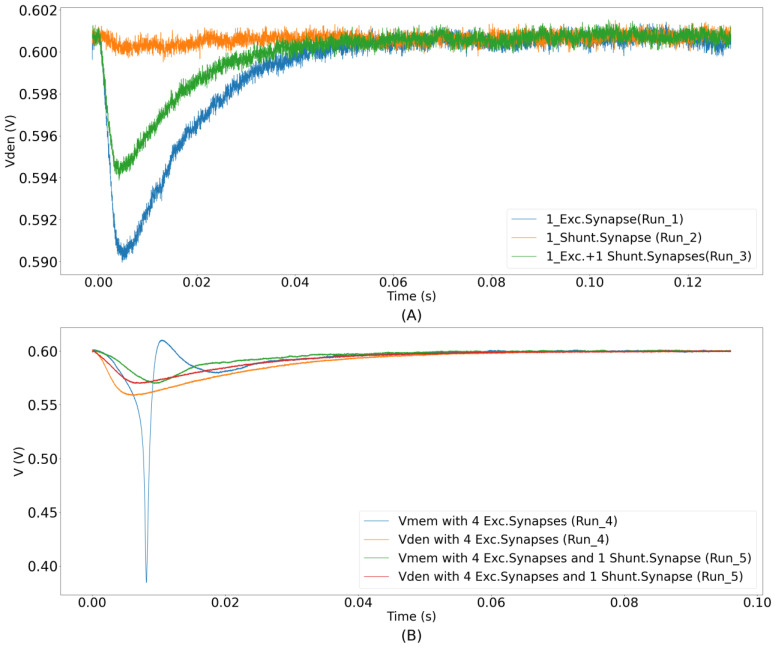
Demonstration of shunting inhibition. (**A**) Three traces of dendritic membrane potentials corresponding to Runs 1, 2, and 3, respectively. Each trace was measured ten times. The figure shows the moving average data of three single traces plotted with a time window of 50 µs. Depolarization reduces when both excitatory and shunting inhibitory synapse circuits are activated together; (**B**) somatic and dendritic membrane potentials for two different runs. Synchronous activation of four synapse circuits causes the soma to spike (blue trace). However, if the additional shunting inhibitory synapse circuit is simultaneously activated, the depolarization is not strong enough and the soma does not spike (green trace).

The shunting inhibition was demonstrated using the unidirectional two-compartment neuron configuration. Of the 256 synapse circuits, 192 were configured to be excitatory (connected via terminal I_syn_exc_), 1 as shunting inhibitory (GABAa-type connected via terminal I_syn_), and 1 was configured as the leak resistor $R_{\mathrm{leak}}$ (connected via terminal I_syn_). The remaining 62 synapse circuits were connected via terminal I_syn_, but were not activated in this demonstration. The synaptic efficacies of all of the synapse circuits were set to the maximum. The resting membrane potential of the soma was set at approximately 600 mV. The synaptic reversal potential ($E_{\mathrm{syn}}$) for the shunting inhibitory synapse circuit was set to 590 mV. For the synapse circuit configured as the resistor $R_{\mathrm{leak}}$, the value of $E_{\mathrm{leak}}$ was set at 590 mV. These values were set based on the relative difference of general electrophysiological values measured from neuronal cells. The average resting membrane potential in neuronal cells is about −70 mV. In our chip, the maximum and minimum voltage supplies were 1 V and 0 V. Furthermore, the soma circuit is designed utilizing PMOS transistors’ characteristics and its spiking behavior is opposite to the convention (See Section 2.3). Due to this, its resting membrane potential is close to 1 V instead of 0 V. It was set to 600 mV in the unidirectional two-compartment configuration, as this value is ideal for the operation of both synapse and soma circuits. Additionally, shunting inhibitory synapses have a reversal potential close to the resting membrane potential (−70 mV), and on average, this value is slightly higher than the resting membrane potential [36,37]. However, as the polarity of the current in our soma circuit is opposite to that of the conventional direction, the value of the synaptic reversal potential (590 mV) was fixed slightly lower than the resting membrane potential. Upon activation, an excitatory synapse generates an EPSP that is shunted if a shunting inhibitory synapse circuit is simultaneously activated. This is because the shunting inhibitory synapse circuit turns on in the right region of the I–V plot in Figure 9B (*E*_syn_ > *V*_mem_), and shunts the EPSP as desired. To demonstrate the shunting inhibition in the circuit experiments, the learning circuitry was deactivated, and the following runs were performed. Initially, only one excitatory synapse circuit was activated by an input spike. In the second run, only the shunting inhibitory synapse circuit was activated, and in the third run, both the excitatory and shunting inhibitory synapse circuits were simultaneously activated. The dendritic membrane potentials for all three cases are plotted in Figure 10A. In the first run, the dendritic membrane potential was strongly depolarized. As expected, in the second run, there was no major change in the dendritic membrane potential. In the third run, the EPSC induced by the excitatory synapse circuit slightly depolarized V_den_. As expected, the EPSP was shunted by the shunting inhibitory synapse circuit, i.e., V_den_ did not depolarize as strongly as in the first case. Each of the three runs above was performed 10 times, and it was observed that shunting inhibition in the second run reduced the amplitude of the EPSP in the first run by an average value of 34.6%, with a standard deviation of 1.65%. Two additional runs were performed using additional excitatory synapse circuits with the same circuit parameters. First, the minimum number of excitatory synapse circuits (four) required to generate a spike were activated synchronously, and next, the same four excitatory synapse circuits were activated along with the single shunting inhibitory synapse circuit. The dendritic (orange and red traces) and somatic (blue and green traces) membrane potentials for both runs are plotted in Figure 10B. As expected, the soma did not spike in the second run, owing to simultaneous activation of the shunting inhibitory synapse. These runs were performed 15 times, and the probability of blocking the soma’s spike by the shunting inhibition was observed as 100%. Synchronous activation of five excitatory synapse circuits was required to overpower the inhibition of a single synapse and for the soma to generate a spike. Instead of using all 192 synapse circuits for the demonstration, we chose fewer circuits, this was done to show visible dendritic depolarization with both single and multiple (four) excitatory synapse circuits. By the appropriate configuration of the circuit’s voltage parameters (that control the amplitude and time constant of excitatory and shunting inhibitory synapse current), the number of synapse circuits can be chosen as desired. The spiking threshold of the soma circuit was approximately 575 mV.

### 3.3. On-Chip Spatiotemporal Spike Pattern Detection

This subsection presents the results of the spatiotemporal spike pattern detection task using adaptive STDP learning, corresponding to Experiment 3 in Table 1. We used the single-compartment neuron configuration with all 256 synapse circuits configured as conductance-based synapses (connected via terminal I_syn_). The goal of this experiment is to demonstrate that the oscillatory nature of the induced synaptic current has no undesired effect on the performance relative to the same experiment with the synapse circuits in the excitatory configuration (connected via the output terminal I_syn_exc_ in Figure 2 and Figure 5). The details and results of the same spike pattern detection task using excitatory synapse circuits are described in another study [30]. The experiments were performed in two groups. In the first (second) group, input spike trains with a spike pattern repetition frequency of 25% (10%) were used. More stochastic spikes are present in the second group (90%) compared to the first group (75%), further increasing the difficulty of the pattern detection task. Upon learning, in successful trials, the neuron only spiked in the presence of the learned spike patterns, and never outside the patterns. The chosen success criterion was a hit rate greater than 98% and zero false alarms in the last 75 s of the run, similar to the criteria used in other studies [28,29,30].

The results of the spike pattern detection task obtained from the experiments with pattern repetition frequencies of 25% and 10% are listed in Table 3. In the former case, 48 out of 50 runs were successful (96% success rate), and in the latter, 44 out of 50 runs were successful (88% success rate). In all these runs, a hit rate of 100% with zero false alarms was obtained in the last 75 s of the run. These runs correspond to Setups 3 and 1 in our previous studies [29] (numerical simulation) and [30] (circuit experiments), respectively. The success rate for 25% (10%) pattern repetition frequency case was 95% (90%) in the former and 96% (90%) in the latter study. Thus, the performance of the proposed conductance-based synapse circuits in the spike pattern detection task is similar to that obtained via the simulations and circuit experiments for the current-based non-oscillatory synapses. The only parameters changed between the two experiments (conductance-based oscillatory synapse circuits and current-based non-oscillatory ones) were $sV_{w}$, the initial value of the synaptic efficacy, and a parameter that controls the current to set the resting potential of the soma circuit. In both cases, the resting membrane potential of the soma circuit was fixed at approximately 850 mV. The initial value of synaptic efficacy was selected to ensure that the spiking frequency of the soma during the initial phase of the run is within the desirable range of 40–200 Hz [29]. The conductance-based synapse circuit requires a relatively higher $sV_{w}$ than the current-based circuit to generate the same current; hence, a higher value was used.

**Figure 11:**
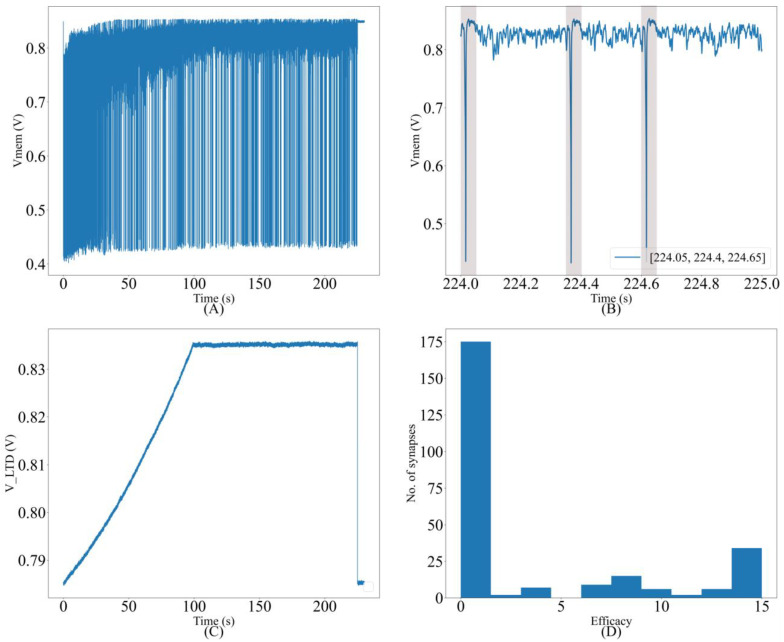
(**A**) Membrane potential of the soma circuit during the run; (**B**) soma circuit’s membrane potential during the last second; it spikes within the shaded 50 ms spike pattern; (**C**) the adaptation of VLTD during learning; (**D**) bimodal distribution of synaptic efficacies after learning.

The value of $sV_{w}$ needed also depends on $V_{\mathrm{dd}\_\mathrm{osc}}$ (see Section 4). These results empirically demonstrate that the oscillatory nature of the synaptic current induced by the proposed synapse circuit has a negligible detrimental effect on the spike pattern detection task. The resting membrane potential of the soma circuit was fixed at approximately 850 mV and the spiking threshold was approximately 700 mV. The initial value of synaptic efficacy was fixed at eight for all synapse circuits. The common voltage parameters of the synapse circuits, i.e.,$\;sV_{w}$, $sV_{t}$, and $E_{\mathrm{syn}},$ were fixed at 315, 160, and 720 mV, respectively. $V_{\mathrm{dd}\_\mathrm{osc}}$ was fixed at 600 mV, and V_post_ was fixed at approximately 600 mV via $V_{\mathrm{CC}\_\mathrm{ref}}$. The evolution of the neuron dynamics for one of the runs with a pattern frequency of 10% is shown in Figure 11. A trace of the membrane potential is shown in Figure 11A. The spiking frequency is high during the initial phase of the run. It decreases as learning progresses and the neuron becomes more selective to the spike inputs. The trace in the last second is magnified in Figure 11B; as expected, the neuron spikes only in the presence of the pattern. The times at which the 50 ms pattern ends are labeled in the bottom-right corner of the figure, and the pattern duration is marked by a box. Figure 11C shows the adaptation of $V_{\mathrm{LTD}}$ during learning, and Figure 11D shows the bimodal distribution of the synaptic efficacies after learning is completed.

During the pattern detection task, the average power consumed by the soma and 256 synapse circuits was measured (from the chip) as under 6 and 25 nW, respectively. These were the average values measured during the initial 50 s of the run, when most of the synapse circuits were active. The average static power consumption of the 256 synapse circuits when they were not activated was less than 450 pW (<2 pW/synapse circuit). The power consumption values reported for the synapse circuit did not include the power consumed by the learning circuitry.

## 4. Discussion and Conclusion

One of the primary goals of neuromorphic computing is to provide real-time or accelerated emulation of neuronal circuitries in dedicated neuromorphic hardware to improve our “understanding” of the brain. This requires neuromorphic implementation of biologically plausible neuronal networks using reduced or multi-compartment neuron configuration. The primary focus of this study was a key component of such a network, a low-power conductance-based synapse circuit. The advantage of this circuit over contemporary analog synapse circuits is its ability to implement shunting inhibition. It was suggested to enhance neuronal computation via the specific non-linear interactions between the excitatory and shunting inhibitory synapses located at different locations on a dendritic tree [18,19,20]. Though the exact mechanisms via which shunting inhibition improves neuronal computation is still unclear, synapse circuits with this capability are critical for the emulation of biologically plausible neuronal networks. The proposed oscillator-based synapse circuit implements a phenomenological conductance-based model of the synapse capable of shunting inhibition. It is suitable for low-power implementations of reduced-or multi-compartment neuron models where the somatic and dendritic compartments are spatially distant. In this study, the simplest possible reduced-compartment neuron model, the unidirectional two-compartment configuration, was chosen to demonstrate the shunting inhibition. 

A natural question on our synaptic circuit is the ill effect of its oscillatory nature. It was shown by circuit experiment that our synaptic circuit had no disadvantage in a most basic biologically plausible neuromorphic computational task. This study does not promote the use of the proposed synaptic circuit over current-based excitatory synapse circuit in single-compartment point neuron configuration because the current-based excitatory synapse circuits have much lower dynamic power consumption (approximately one order of magnitude lower) [30] compared to the proposed circuit. In the spike pattern detection experiment, the conductance-based synapse circuit was intentionally configured to act as an excitatory current-based synapse in a point neuron configuration to demonstrate that its oscillatory nature has a negligible effect on neuronal information processing in the task, and the measured results empirically support this. This single-compartment point neuron model (without shunting inhibition) was selected because we could not find a clear computational model that exploits the shunting inhibition.

The ideal operating frequency of the ring-oscillator-type circuit in the transconductance stage should range between 2 and 25 KHz, corresponding to synaptic efficacies of 1 and 15, respectively. The minimal value of 2 KHz was chosen because it is sufficiently higher than the maximum spike frequency of neuronal cells, and its period (500 µs) is not too close to the minimum synaptic decay time constant of AMPA synapses (~3 ms). A high operating frequency leads to high fidelity in the induced synaptic current, but at the cost of higher dynamic power consumption. Hence, a trade-off must be made between the operating frequency and power consumption of the circuit. It can be made based on the desired application. In this study, the voltage parameters for the synapse circuits were tuned manually without any consideration to minimize the power consumption. In the future, the minimum oscillator frequency required for the spike pattern detection task will be explored via numerical simulations.

The conductance implemented by the proposed synapse circuit between $V_{\mathrm{mem}}$ (or $V_{\mathrm{den}}$) and $E_{\mathrm{syn}}$ is non-linear, with an exponential dependence. The circuit has an exponentially lower conductance for $E_{\mathrm{syn}}$ < $V_{\mathrm{mem}}$ (left region of the I–V plot in Figure 9B) than for $E_{\mathrm{syn}}$ > $V_{\mathrm{mem}}$. The conductance can be increased by decreasing $\;V_{\mathrm{dd}\_\mathrm{osc}}$ relative to $E_{\mathrm{syn}}$ (Figure 9B). Although this non-linear conductance is not detrimental to implementing the shunting inhibition phenomenon (the circuit operates in the right region of the I–V plot in Figure 9B), a more linear relationship would better fit the phenomenological conductance-based synapse model (Equation (1)). Any circuits in which the oscillator’s behavior is controlled without disturbing its power terminals will be explored in the future with the goal of removing the exponential non-linearity.

The static power consumption of the proposed synapse circuit was measured as 1.64 pW, resulting in 1.64 nW for 1000 synapse circuits. We could not find the static power consumption metric for comparison with low-power current-based synapse circuits such as LDI and DPI synapse circuits [26,32]. In point neuron circuits with linear synapse models, the static power consumption is not important, because a synapse circuit can be shared by multiple synaptic inputs. However, for a multi-compartmental implementation in which the shunting inhibition synapses play important roles, minimizing the static power consumption is important, because many synapse circuits have to be implemented at different physical locations according to the desired spatial configuration of the neuron. Hence, minimizing the static power consumption of a single circuit is an important design constraint. The dynamic power consumption of this circuit is comparatively higher. In the spike pattern detection task, the 256 conductance-based synapse circuits (configured to act as excitatory synapses) consumed less than 26 nW of power (measured from the chip), and in the same task using current-based excitatory synapse circuits, the power consumption was less than 2.5 nW [30]. The power consumed by conductance-based synapse circuits is significantly higher, but can be reduced by using a smaller value of $\;V_{\mathrm{dd}\_\mathrm{osc}}$. The circuit operates reliably across all process corners for values of $V_{\mathrm{dd}\_\mathrm{osc}}$ > 450 mV. When $V_{\mathrm{dd}\_\mathrm{osc}}$ = 450 mV, a much smaller $sV_{w}$ is required to induce the same current. In the spike pattern detection experiment, with $V_{\mathrm{dd}\_\mathrm{osc}}$ = 450 mV (reduced from 600 mV) and $sV_{w}$ = 215 mV (reduced from 315 mV) and all other parameters remaining unchanged, the power consumed by 256 synapse circuits was measured to be less than 5 nW. However, using a smaller $\;V_{\mathrm{dd}\_\mathrm{osc}}$ of 450 mV increased the static current flowing out from $E_{\mathrm{syn}}$ to $V_{\mathrm{mem}}$, and thus increased the overall static power consumption. In our experimental setup, no provision was present to measure the current sourced by $\;E_{\mathrm{syn}}$. As such, this increase in the static current (and thus the change in the static power) was evaluated using the Spectre simulator. For a synapse circuit, the static current flowing from $E_{\mathrm{syn}}$ to $V_{\mathrm{mem}}$ increased from approximately 20 fA to 1.41 pA when $V_{\mathrm{dd}\_\mathrm{osc}}$ was reduced from 600 mV to 450 mV. For 256 synapse circuits, this would amount to approximately 362 pA, and the power consumption would increase by less than half a nano watt. Thus, the power consumption of the synapse circuit can be minimized at the expense of a relatively higher static current (from $E_{\mathrm{syn}}$ to V_mem_), whose effect on the membrane potential can be compensated for at the dendritic level. The bulk terminal V_bulk_ provides additional control over the current induced (both static and dynamic) by the synapse circuits and can be used to minimize the static current. For a multi-compartment neuron implementation with a large number of synapses, minimizing the static current flowing into or out of the synapse circuits is important for minimizing the overall power consumption. At the dendritic level, every node in the compartmental model would require a wide-range transconductance circuit to compensate for the static current flowing into that node (to maintain the resting membrane potential of that compartment). The higher the current, the higher the power consumption of the transconductance circuits.

In the single-compartment configuration, power consumption can be further reduced by improving the design of the current conveyor circuit. The current-conveyor circuit induced noise in the soma circuit. To minimize this effect, the power terminals of its output branch were fixed at 949 mV and 50 mV, instead of the ideal values (1 V and 0 V, respectively), and the resting membrane potential of the soma circuit was maintained at 850 mV (150 mV higher than the spiking threshold). To account for this change, a relatively higher $sV_{w}$ value (315 mV) was required. By improving the design of the current conveyor circuit, a smaller value of $sV_{w}$ can be used, thereby reducing the power consumption of the circuit. The dynamic power consumption of this circuit will still be higher than that of current-based synapse circuits. The additional functionality of the proposed circuit comes at the cost of a relatively high power consumption.

As an interface, the unidirectional resistor consumes much less power than the current conveyor circuit (approximately two orders of magnitude lower); thus, the unidirectional two-compartment neuron model can be more efficient than the widespread single-compartment neuron model. The experimental results of the spike pattern detection task using the unidirectional two-compartment model and its comparison with the single-compartment configuration will be presented in a future study. The neuron circuit was fabricated in a relatively older TSMC 250 nm technology node, but all the circuits presented in the study are compatible with lower-technology nodes; 250 nm was chosen because of its availability and financial constraints. A 28 nm fully depleted silicon-on-insulator (FD-SOI) technology will be used for future work.

## Figures and Tables

**Table 1:** Summary of the experiments performed in this study.

Exp. #	Demo.	Neuron Configuration			Synapse Circuits Configuration (Terminal Name)		
		Model	Resting V_mem_	Spiking Threshold	Excitatory (I_syn_exc_)	Conductance-Based Synapse (I_syn_)	Conductance-Based Resistor (I_syn_)
1	Synapse circuit characteristics	-	-	-	-	-	-
2	Shunting inhibition	Unidirectional two-compartment	600 mV	575 mV	192	1	1
3	Spike pattern detection	Single-compartment	850 mV	700 mV	-	256	-

**Table 2:** Synapse circuit’s voltage parameters used for measurements. Synaptic efficacy was set to maximum value for both measurements.

Measurements	$sV_{w}$	$sV_{t}$	$V_{\mathrm{dd}\_\mathrm{osc}}$	$E_{\mathrm{syn}}$	$V_{\mathrm{mem}}$
Characteristics of the synapse circuit	370 mV (340 mV)	80 mV	600 mV (500 mV)	500 mV to 700 mV	600 mV
Single synapse power consumption	315 mV	160 mV	600 mV	700 mV	600 mV

**Table 3:** Results and comparative performance in the spike pattern detection task.

	Simulation Results with Non-Oscillatory Current-Based Synapses [29]	Experimental Results with Non-Oscillatory Current-Based Synapses [30]	This Study with Oscillatory Conductance-Based Synapses
Success rate with 25% pattern frequency	95%	96%	96%
Success rate with 10% pattern frequency	90%	90%	88%

## Data Availability

The procedure used to generate data used in this study is described in the article.
